# Supplementary material for: Coexistence of ferroelectricity and antiferroelectricity in 2D van der Waals multiferroic
Source: Nat Commun. 2024 Oct 4;15:8616. doi: 10.1038/s41467-024-53019-5 (PMC11452644; doi:10.1038/s41467-024-53019-5)
Supplement: Supplementary file 1 — Supplementary Information [file 41467_2024_53019_MOESM1_ESM.pdf]

Supplementary Materials for

Coexistence of ferroelectricity and antiferroelectricity in 2D van der Waals multiferroic

Yangliu Wu[1], Zhaozhuo Zeng[2], Haipeng Lu[1], Xiaocang Han[3], Chendi Yang[4], Nanshu Liu[5], Xiaoxu Zhao[3], Liang Qiao[6], Wei Ji[5] ✉, Renchao Che[4], Longjiang Deng[1] ✉, Peng Yan[2] ✉ and Bo Peng[1] ✉

[1]National Engineering Research Center of Electromagnetic Radiation Control Materials and Key Laboratory of Multi Spectral Absorbing Materials and Structures of Ministry of Education, School of Electronic Science and Engineering, University of Electronic Science and Technology of China, Chengdu 611731, China

[2]School of Physics and State Key Laboratory of Electronic Thin Films and Integrated Devices, University of Electronic Science and Technology of China, Chengdu, 610054, China

[3]School of Materials Science and Engineering, Peking University, Beijing 100871, China

[4]Laboratory of Advanced Materials, Department of Materials Science, Collaborative Innovation Center of Chemistry for Energy Materials(iChEM), Fudan University, Shanghai 200433, China

[5]Beijing Key Laboratory of Optoelectronic Functional Materials & Micro-Nano Devices, Department of Physics, Renmin University of China, Beijing 100872, China

[6]School of Physics, University of Electronic Science and Technology of China, Chengdu 611731, China

✉ To whom correspondence should be addressed. Email address: bo_peng@uestc.edu.cn; yan@uestc.edu.cn; denglj@uestc.edu.cn; wji@ruc.edu.cn

The PDF file includes:
Supplementary Text
Supplementary Note 1-3
(Supplementary Fig. 1 to 15)

Supplementary Text

Density functional theory calculations. Density functional theory (DFT) calculations were performed using the generalized gradient approximation (GGA) for the exchange–correlation potential, the projector augmented wave (PAW) method[1,2], and a plane-wave basis set as implemented in the Vienna *ab initio* simulation package (VASP)[3,4]. A kinetic energy cutoff of 700 (650) eV for the plane waves was used for structural optimization (calculations on the relative energies). The on-site Coulomb interaction was considered with a U value of 4.2 eV and a J value of 0.8 eV for Ni 3d orbitals, according to the literature[5,6] and our energy test calculations. Spin–orbit coupling (SOC) was considered in all total energy calculations. We constructed a 7×1 supercell to model the spiral orders for trilayer NiI_2. All atoms, lattice volumes, and shapes in each supercell were allowed to relax until the residual force on each atom was less than 0.01 eV/Å. A vacuum layer over 20 Å in thickness in the z direction was adopted to eliminate interactions among image layers. Grimme's semiempirical D3 scheme[7] for dispersion correction was employed to describe the vdW interactions in combination with the Perdew–Burke–Ernzerhof functional (PBE-D3)[8]. More detailed calculation methods can be found in our previous work[9].

Atomistic spin model simulations. The atomistic spin model simulations are performed by numerically solving the Landau-Lifshitz-Gilbert (LLG) equation. The atomic scale LLG equation can be read $-\frac{1+\alpha^2}{\gamma}\frac{\partial S_i}{\partial t} = S_i \times H_i + \alpha S_i \times (S_i \times H_i)$, where the S_i is the reduced magnetization of the ith atom, γ is the gyromagnetic ratio, α is the Gilbert damping constant, $H_i = -\frac{\partial \mathcal{H}}{\mu_s \partial S_i}$ is the effective field, $\mathcal{H}$ is the Hamiltonian, and the magnetic moment μ_s is set to $2\mu_B$ for each Ni^{2+}, respectively. In the NiI_2, the Hamiltonian with the Kitaev and biquadratic interactions can be expressed as

$$\mathcal{H} = -\frac{1}{2}\left[\sum_{\langle i,j\rangle_n} J_n S_i \cdot S_j + \sum_{\langle i,j\rangle_n} J_n^{\perp} S_i \cdot S_j + \sum_{\langle i,j\rangle_1} K S_i^{\lambda} S_j^{\lambda} + \sum_{\langle i,j\rangle_1} B\left(S_i \cdot S_j\right)^2 + 2\sum_i A_z S_z^2\right] \quad (1)$$

where n = 1, 2, 3 represents the nth nearest neighbor coupling, J_n and $J_n^{\perp}$ are the intra- and interlayer Heisenberg exchange parameters, K and B are the Kitaev and biquadratic interaction parameters, λ is the basis of the Kitaev interaction corresponding to $S_i^{\lambda} = S_i \cdot \lambda$, A_z is the single-ion anisotropy parameter, respectively. Hence, the effective field can be derived

$$H_i = \frac{1}{2\mu_s}\left[\sum_{\langle i,j\rangle_n} J_n S_j + \sum_{\langle i,j\rangle_n} J_n^{\perp} S_j + \sum_{\langle i,j\rangle_1} K\left(S_j \cdot \lambda\right)\lambda + 2\sum_{\langle i,j\rangle_1} B\left(S_i \cdot S_j\right) S_j + \right.$$

$4 \sum_i A_z (S_i \cdot e_z) e_z]$ (2)

The periodic boundary condition (PBC) in *a* and *b* direction is adopted. Following the previous work[9], the intralayer Heisenberg exchange coupling is J_1 = 3.36 meV, J_2 = 0.07 meV, J_3 = -3.06 meV, and the interlayer Heisenberg exchange coupling is $J_1^{\perp}$ = 0.04 meV, $J_2^{\perp}$ = -1.29 meV, $J_3^{\perp}$ = -0.23 meV, respectively. The Kitaev interaction parameter K = 2.42 meV, biquadratic interaction parameter B = 0.37 meV, and single-ion anisotropy parameter A_z = 1.58 meV are adopted. The basis of Katiev interaction can be described as $\lambda_{i-1,j} = \lambda_{i+1,j} = \left(0, -\frac{\sqrt{2}}{\sqrt{3}}, \frac{1}{\sqrt{3}}\right)$, $\lambda_{i,j-1} = \lambda_{i,j+1} = \left(\frac{1}{\sqrt{2}}, \frac{1}{\sqrt{6}}, \frac{1}{\sqrt{3}}\right)$, and $\lambda_{i-1,j-1} = \lambda_{i+1,j+1} = \left(-\frac{1}{\sqrt{2}}, \frac{1}{\sqrt{6}}, \frac{1}{\sqrt{3}}\right)$, respectively. A random state is performed as an initial state. To quickly reach the equilibrium state, the Gilbert damping constant α is set to 0.5 and the time interval of iteration is 1 fs. Utilizing the KNB model[10], the electric polarization texture resulting from the magnetic texture can be expressed as $\mathbf{P}_{ij}=\sigma \mathbf{n}_{ij} \times (\mathbf{S}_i \times \mathbf{S}_j)$, where $\mathbf{P}_{ij}$ represents the electric polarization, $\mathbf{n}_{ij}$ denotes a unit vector linking the two neighboring spins $\mathbf{S}_i$ and $\mathbf{S}_j$, and σ is a scalar parameter dependent on the details of the electronic level structure.

KAI model. The switching dynamics of ferroelectric materials is typically described by Kolmogorov-Avrami-Ishibashi (KAI) model. The normalized change in electric polarization can be expressed by the compressed exponential function[11]

$$\Delta P(t)/2P_r = 1 - \exp\left[-\left(\frac{t}{\tau}\right)^n\right]$$

$$-\ln(1 - \Delta P(t)/2P_r) = \left(\frac{t}{\tau}\right)^n \qquad (3)$$

where τ is a characteristic switching time. In the conventional KAI model, the index n depends on the dimensionality of the domains.

The $\Delta P(t)$, P_r and corresponding time t extract from frequency (f) dependence of *P*-*E* loops at different electric fields and temperatures. The $-\ln(1-\Delta P(t)/2P_r)$ as a function of corresponding time ($t = 1/4f$) at various magnetic fields are presented in Fig. 4f and 4g. The solid lines are fits to the compressed exponential function of Eq. (3). An excellent agreement is observed. The Avrami index n and the switching time τ are obtained from the fitting parameters.

Magnetic control ferroelectric switching at 10 K. In our experiment, the magnetic control of ferroelectric switching was measured at a fixed electric field of 0.74 MV/cm. It is shown that the $-\ln(1-\Delta P(t)/2P_r)$ as a function of time is linear, as presented in Fig. 4f and 4g. These results are characterized by an Avrami index of 1, which indicate that

the ferroelectric polarization domains grow in a 1D fashion. The magnetic field exhibits a deceleration effect on the ferroelectric switching, which is consistent with the observed shift of current peaks in Fig. 4e and Supplementary Fig. 14.

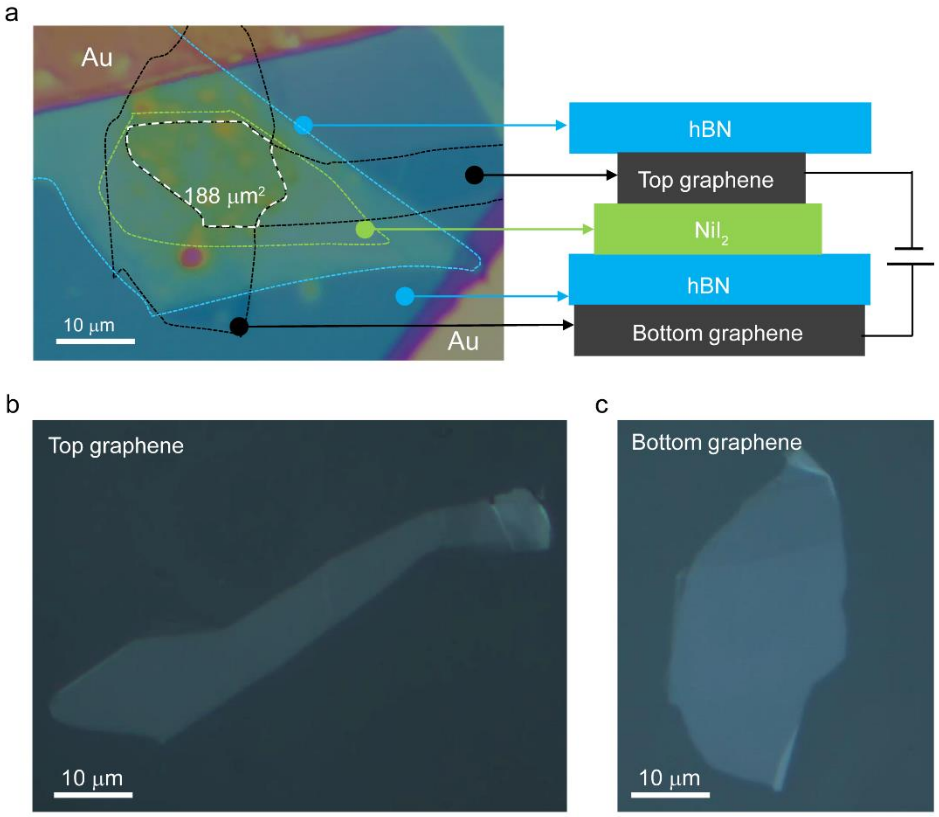

Supplementary Fig. 1 | Trilayer NiI_2 device. a Optical micrograph (left) and schematic side view (right) of a trilayer NiI_2 sample on hBN substrate and covered by graphene and another hBN on the top. The white dashed line indicates the area of trilayer NiI_2 between two graphene electrodes, which were contacted with Au electrodes through a PDMS dry transfer method and further directly connected with ferroelectric tester (Precision Premier II: Hysteresis measurement) and voltage source meter. **b, c** The optical micrograph of top and bottom graphene as electrodes. The area of NiI_2 sandwiched by top and bottom graphene electrodes is estimated to be ~188 μm^2 by grid segmentation method.

Supplementary Note 1. The detection of Raman modes is closely related to the Raman tensor and the polarization configurations of the incident and scattered light. According to the Raman selection rule, the Raman scattering intensity is proportional to $|\boldsymbol{\sigma}^{\dagger}_{s} \cdot \tilde{R} \cdot \boldsymbol{\sigma}_{i}|^2$. For rhombohedral (R$\bar{3}$m) NiI_2, the A_{1g} and E_g modes are active. The Raman intensities of A_{1g} (124.7 cm^{-1}) and E_g (76 cm^{-1}) in circularly polarized configurations of σ+σ+ and σ-σ- channels are

$$I_{A1g}^{\sigma-\sigma-} = I_{A1g}^{\sigma+\sigma+} \propto a^2$$

$$I_{Eg}^{\sigma-\sigma-} = I_{Eg}^{\sigma+\sigma+} \propto 0$$

Hence, in the σ+σ+ and σ-σ- channels, only A_{1g} is detectable, whereas E_g is not detectable, aligning well with the Raman spectra (Fig. 1d).

Alternatively, in the linearly polarized configurations of XX and XY channels, the Raman intensities of the A_{1g} *and* E_g modes are

$$I_{A1g}^{XX} \propto a^2 \qquad I_{A1g}^{XY} = 0$$

$$I_{Eg}^{XX} \propto c^2 \qquad I_{Eg}^{XY} \propto c^2$$

Therefore, the E_g mode is observed in both XX and XY channels, while the A_{1g} mode is only identifiable in the XX channel. This observation is consistent with the results obtained in our Raman experiments (Supplementary Fig. 2), which are also consistent with previous researches[12].

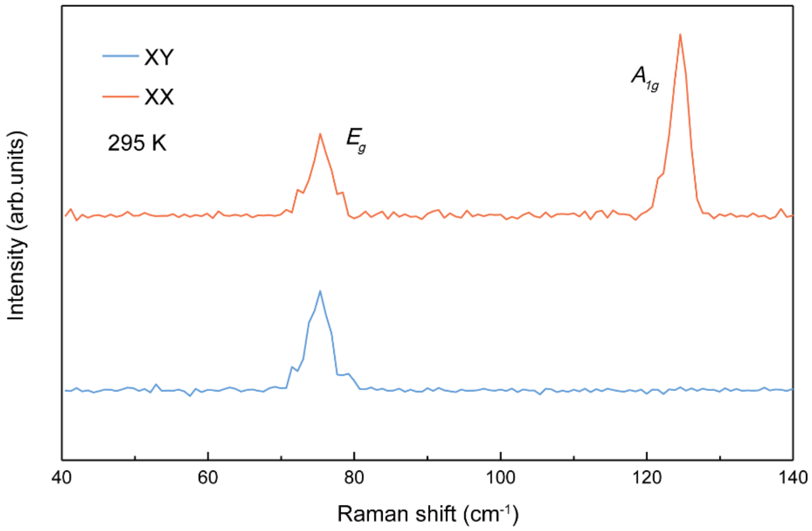

Supplementary Fig. 2 | Raman spectra of trilayer NiI_2 were obtained in channels XX and XY at room temperature.

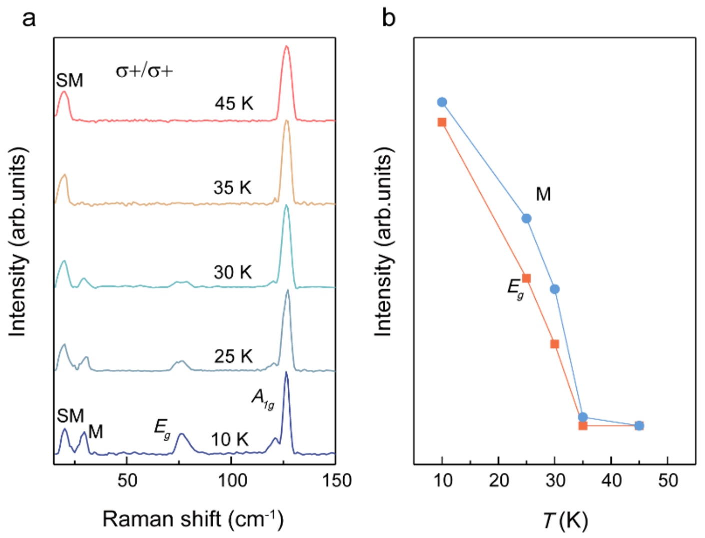

Supplementary Fig. 3 | Temperature-dependent Raman results of 3L NiI_2 in σ+σ+ channel. a Circular polarization resolved Raman spectra of 3L NiI_2 with temperature varying from 10 K up to 45 K. **b** Temperature-dependent Raman peak intensity of the E_g and M modes (magnon), respectively. The E_g feature emerge due to the magnon-phonon coupling induced by magnetic orders. The Raman results suggest that the magnetic transition takes place at approximately 35 K.

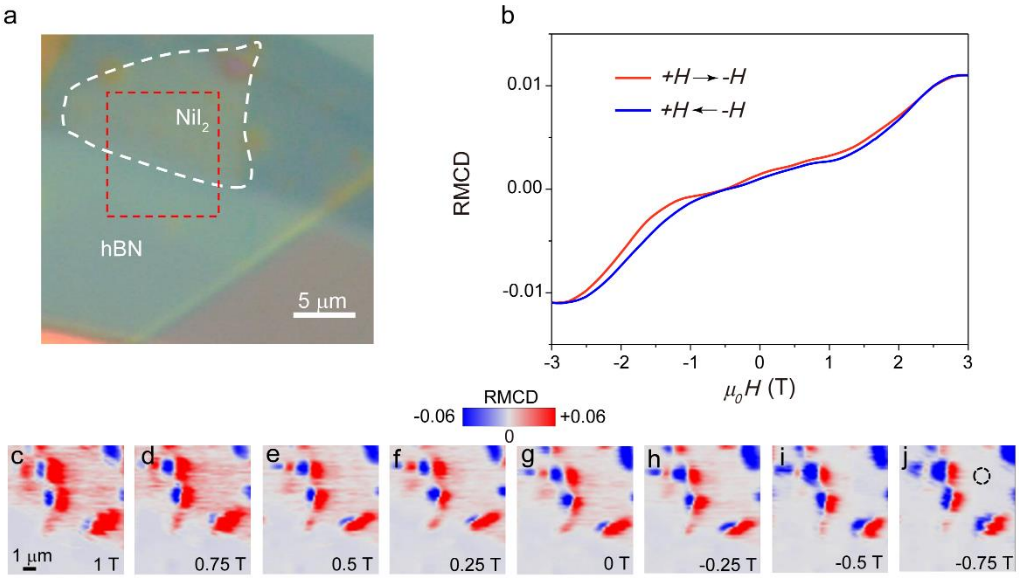

Supplementary Fig. 4 | Magnetic texture in few-layer NiI_2 encapsulated by hBN at 10K. **a** Optical micrograph of another few-layer NiI_2 put on hBN substrate and further covered by another hBN on the top. The white and red dashed-line box represents the profile of the NiI_2 flake and the area of RMCD maps. **b** The RMCD curves sweeping between +3 T and -3 T at 10 K, suggesting a non-collinear antiferromagnetism. The result was collected from the area enclosed by the black circle in **j**. The blue (red) curve corresponds to increasing (decreasing) field. **c-j** Polar RMCD maps at 10 K, taken at selected out-of-plane magnetic field.

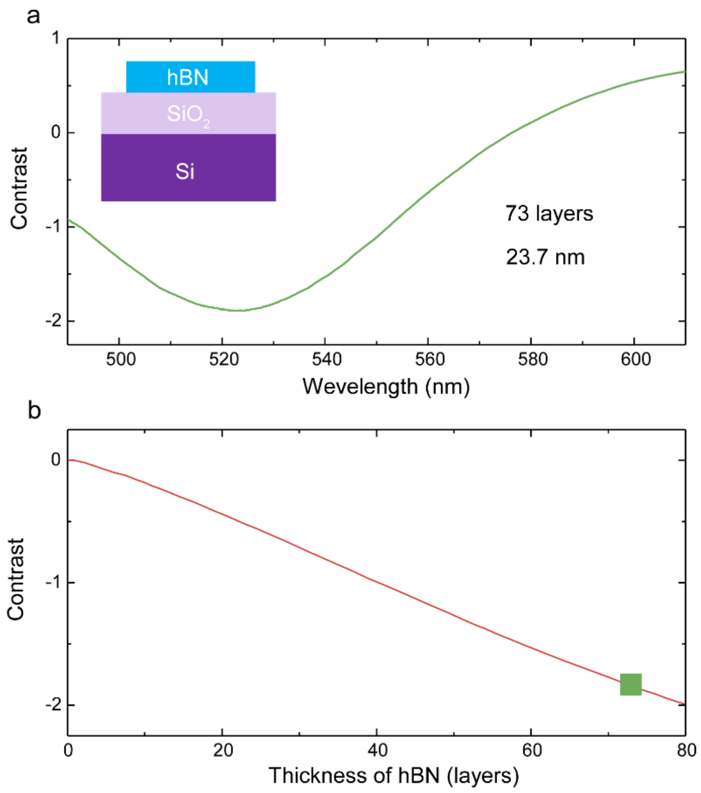

Supplementary Fig. 5 | Thickness of hBN in device. a The optical contrast of the bottom hBN on a SiO_2/Si substrate as a function of the wavelength of light. **b** The linear dependence of the optical contrast at 516 nm on the thickness of the hBN nanoflake between 1 and 80 layers[13]. The optical contrast at 516 nm indicates that the bottom hBN is approximately 73 layers, as shown in the light green square.

Supplementary Note 2. Supplementary Fig. 6a shows the leakage current from the Gr/NiI_2/Gr and Gr/NiI_2/hBN/Gr devices at 10 K, demonstrating that the introduction of the insulating hBN layer effectively reduces the leakage current to near zero. However, significant leakage current occurs in the Gr/NiI_2/Gr device. Upon calculation, the resistance of the Gr/NiI_2/hBN/Gr device is determined to be 3.2×10^{11} ohms, significantly higher than the resistance of the Gr/NiI_2/Gr device, which is only 840 ohms. Supplementary Fig. 6b illustrates the *P-E* behavior of the Gr/NiI_2/Gr device at 10 K and 1.3 Hz, displaying a ball-like loop characteristic of resistive leakage[14,15]. Additionally, the *I-E* curve of the Gr/NiI_2/Gr device in Supplementary Fig. 6c exhibits distinct linear current behavior, rather than ferroelectric characteristics, indicating that the leakage current suppresses the ferroelectric behavior of few-layer NiI_2. Thus, a high-quality insulating dielectric is crucial for assessing ferroelectricity. The hBN flake serves as an effective insulating layer to mitigate substantial leakage current and enable the detection of ferroelectric features, as reported in previous studies[16,17].

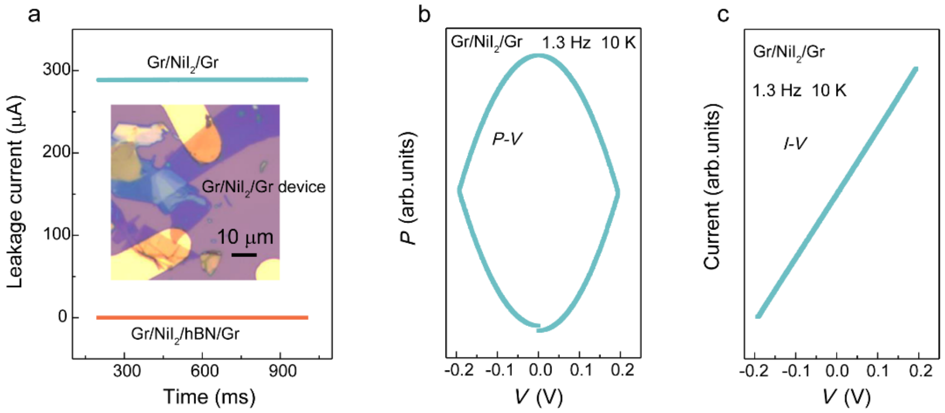

Supplementary Fig. 6 | Reduce the leakage current using hBN. a Leakage-time characteristics measured at 10 K for the Gr/NiI_2/Gr and Gr/NiI_2/hBN/Gr devices. The insert shows the optical photograph of the Gr/NiI_2/Gr device. **b, c** *P-V* and *I-V* loops at 10 K from the Gr/NiI_2/Gr device.

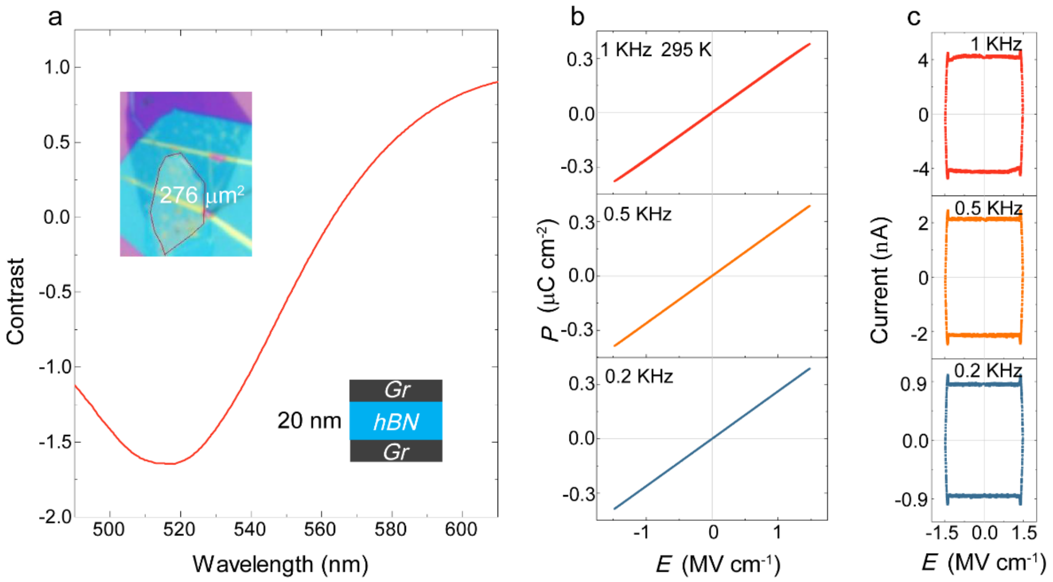

Supplementary Fig. 7 | Background signals from hBN flake. a The optical contrast of a pristine hBN flake composing a graphene/hBN/graphene device to check the electric properties of pristine hBN as control experiments. The selected pristine hBN flake is around 21 nm thickness, close to that in trilayer NiI_2 device. The insets show the optical microscopy image and device schematic. **b-c** The *P-E* and *I-E* loops of pristine hBN devices at various frequency. The pristine hBN shows a linear behavior with electric field, suggesting pristine hBN is an excellent insulator without ferroelectric polarization.

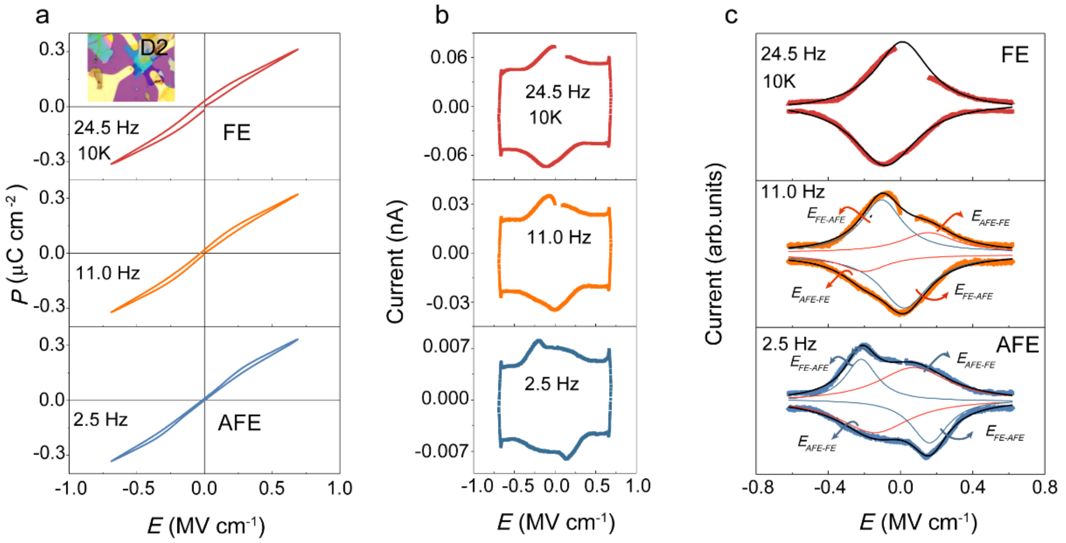

Supplementary Fig. 8 | Coexistence of FE and AFE orders in a few layers NiI_2. a, b Raw *P-E* and *I-E* loops as a function of the frequency of applied electric field at 10 K (Device 2). **c** Corresponding *I-E* curves of (**b**) after subtracting the current background, fitted by Lorentz function. As decreasing frequency, a single-hysteresis loop of FE features along with a pair of opposite switching current peaks, evolves to a characteristic double-hysteresis loop of AFE polarization, typically with two pairs of switching current peaks and decreasing P_r. This suggests that an evolution from FE to AFE takes place.

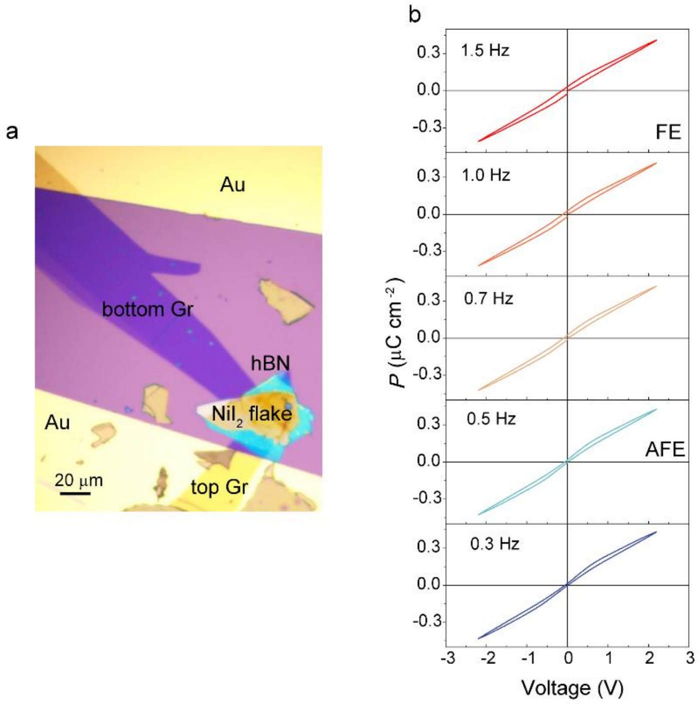

Supplementary Fig. 9 | Coexistence of FE and AFE orders in the bulk-like NiI_2. a The optical photograph of the Gr/bulk NiI_2/hBN/Gr device. **b** *P*-*E* loops at various frequencies obtained from the bulk-like NiI_2 device at 10 K.

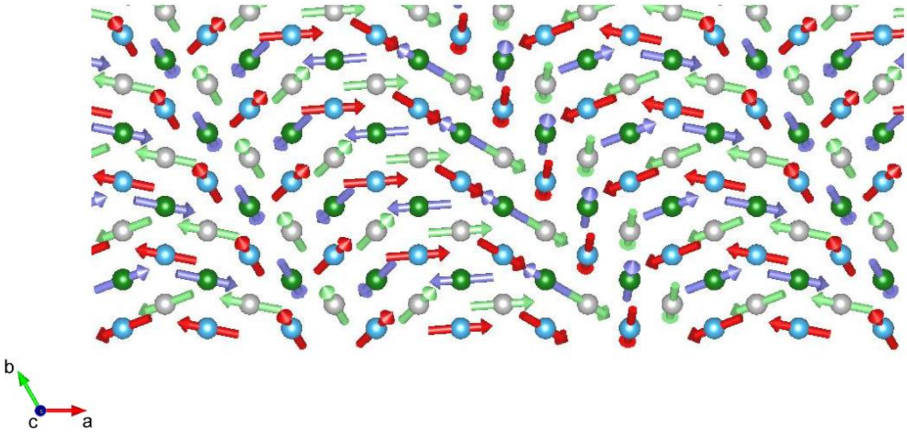

Supplementary Fig. 10 | Top views of the magnetic order of trilayer NiI_2. The blue, green, and gray balls represent Ni atoms in the first, second, and third layers, respectively, while the arrows indicate the spins of Ni atoms.

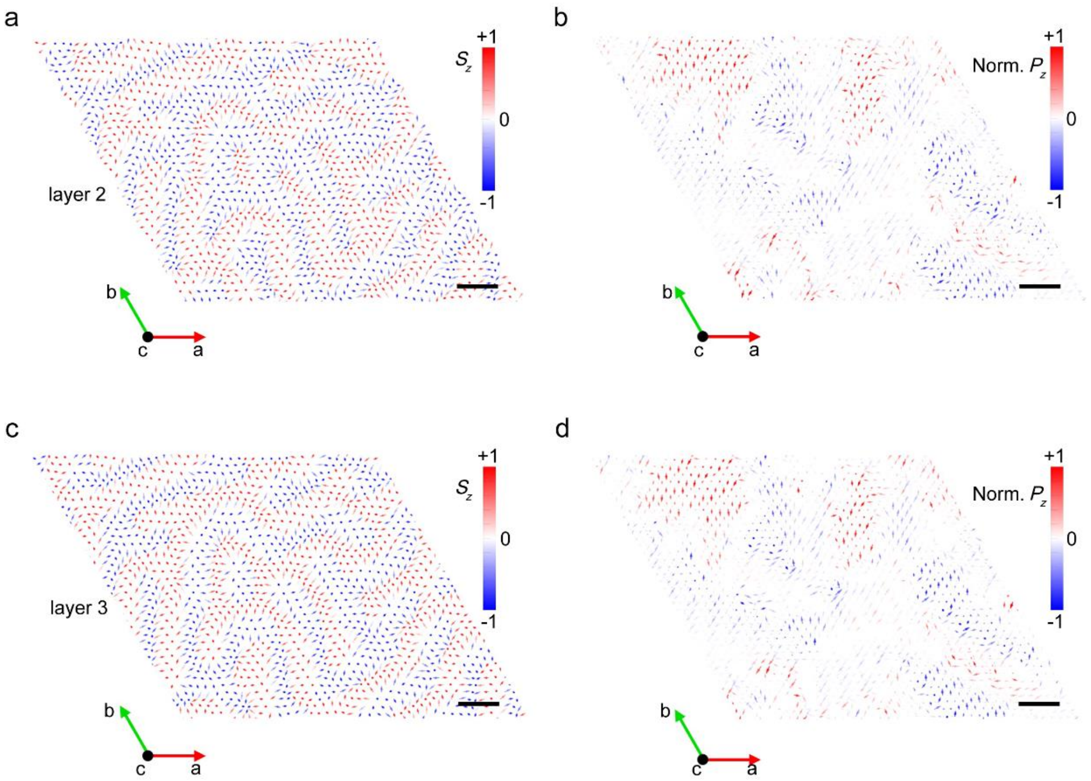

Supplementary Fig. 11 | The Origin of the coexisting ferroelectric and antiferroelectric. a, c Spin textures and corresponding ferroelectric dipole textures (**b, d**) from theoretical simulations. Spins and ferroelectric dipoles are represented by arrows, with red and blue colors indicating positive and negative values of the out-of-plane component. The scale bars are 2 nm.

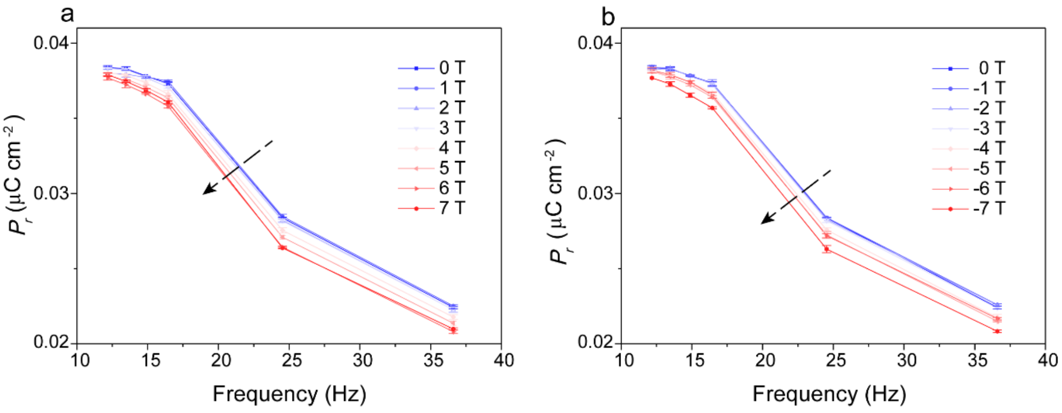

Supplementary Fig. 12 | Magnetic control of ferroelectricity in trilayer NiI_2 device. **a** The frequency-dependent remanent polarization P_r curves in the range of 0 to 7 T magnetic field. **b** The frequency-dependent remanent polarization P_r curves in the range of 0 to -7 T magnetic field. For all relevant panels error bars represent mean ± standard error of the mean.

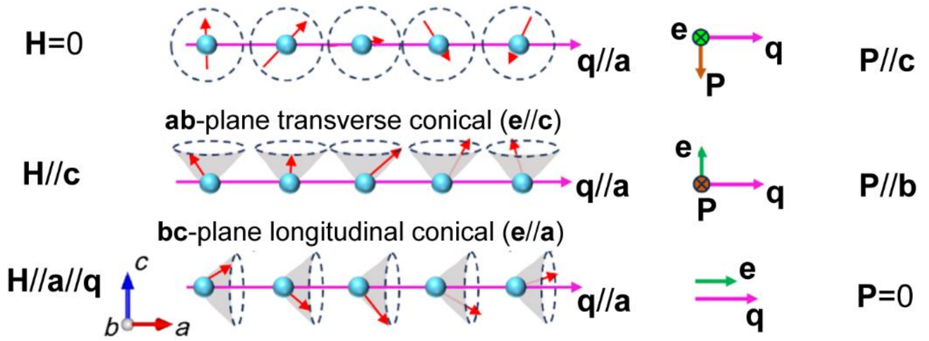

Supplementary Fig. 13 | Magnetic control of electric polarization in spiral magnets. Schematics of **ac**-plane transverse spin spiral with propagation vector **q** // **a** (top panel), which induces out-of-plane ferroelectric polarization **P** // **c**. Application of magnetic fields **H** // **c** (middle panel) and **H** // **a** (bottom panel) to stabilize the transverse and longitudinal spin conical, respectively, along with magnetization **M** // **H,** and the ferroelectric polarization is expected to **P** // **a** and **P**=0 through the KNB model.

Supplementary Note 3. The observed background current (Fig. 4e and Supplementary Fig. 14) is identified as displacement current rather than conduction current. Displacement current (*I*) is defined as the time rate of change of the electric displacement flux passing through a surface, a concept initially introduced by Maxwell. The displacement current density vector is symbolized as $\mathbf{j}=\partial\mathbf{D}/\partial t$. Considering the relationship $\mathbf{D}=\varepsilon_0\mathbf{E}+\mathbf{P}$, where **E** represents the electric field vector and **P** denotes the polarization vector in dielectric materials, the displacement current density **j** is expressed as $\mathbf{j}=\varepsilon_0\partial\mathbf{E}/\partial t + \partial\mathbf{P}/\partial t$. Consequently, for dielectrics polarized linearly with the electric field, such as hBN, the background current remains constant and escalates with the frequency increase (i.e., the rate of change of the electric field over time; Supplementary Fig. 7c). In stark contrast, in ferroelectric materials, the ferroelectric polarization undergoes a sudden reversal at the critical electric field (large $\partial\mathbf{P}/\partial t$), leading to a substantial current peak (Figs. 3b and c). In Fig. 4e, the magnetic field solely influences the current peak, suggesting that the magnetic field governs the ferroelectric polarization reversal with the critical electric field in NiI_2. However, the background current does not participate in the ferroelectric polarization reversal; hence, the magnetic field does not impact the background current. The experimental results derived from the graphene/NiI_2/hBN/graphene device demonstrate that the background current arises from the capacitor's charging and discharging process, rather than the conductive tunneling current through the NiI_2 layer. Therefore, the magnetic field-controlled ferroelectric measurements do not involve magnetoresistance.

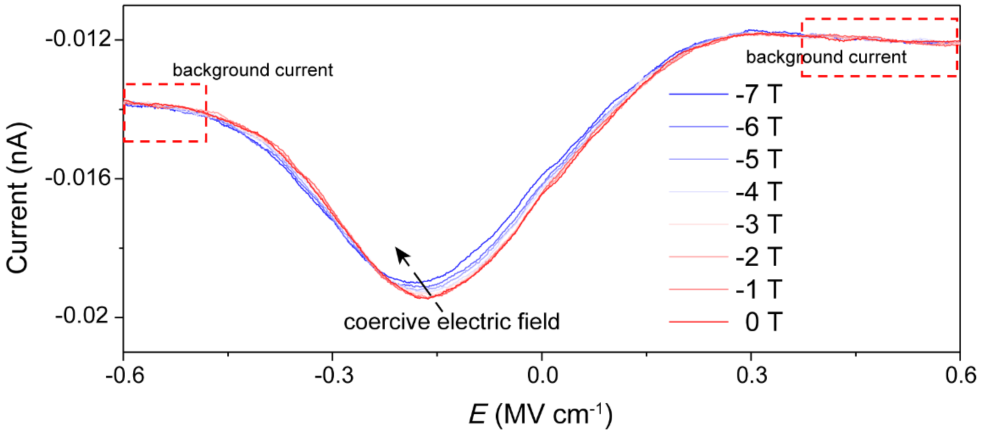

Supplementary Fig. 14 | Magnetic control of current peak in trilayer NiI_2 device. The *I-E* curves at different magnetic fields ranging from 0 to -7 T. The black dashed arrows indicate the shift in current peaks with increasing magnetic field, while the red dashed boxes highlight the background current that remains unchanged as the magnetic field increases. The clear decrease in the current peak, along with a rise in the coercive electric field, is linked to the increased magnetic field.

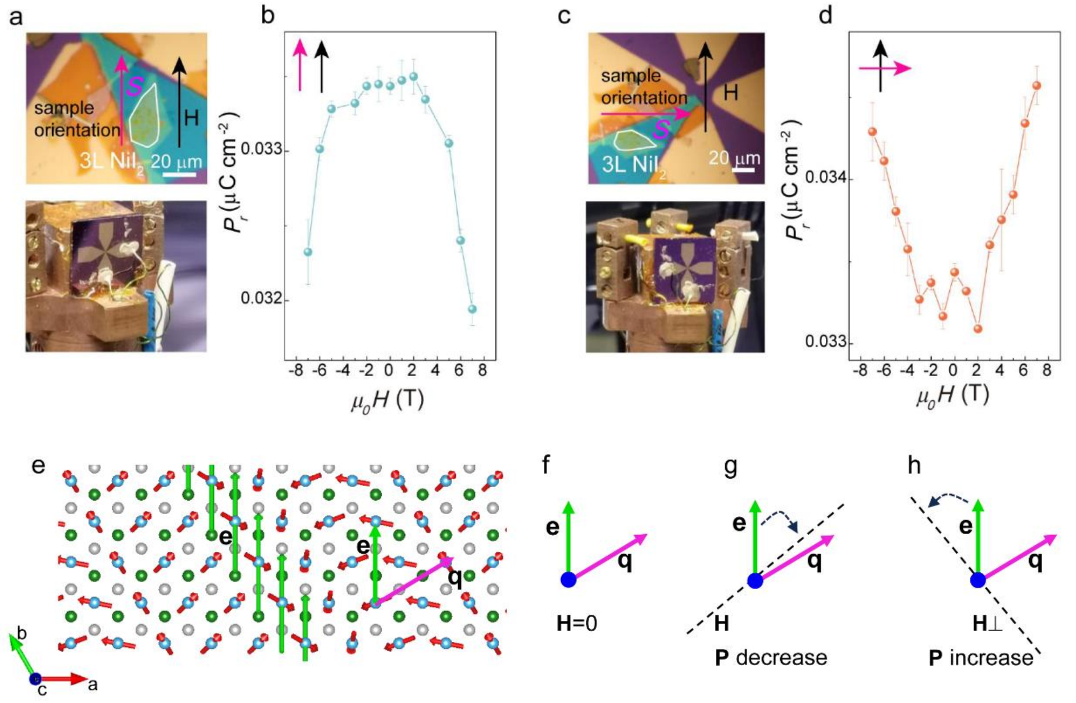

Supplementary Fig. 15 | In-plane magnetic control of ferroelectricity in trilayer NiI_2 device. **a, c** Optical photographs of the samples at different geometric configurations where the magnetic field is parallel or perpendicular to *S*-direction (sample orientation). The direction of the magnetic field is fixed, and the parallel and perpendicular configurations are achieved by rotating the sample. In panels **a** and **c**, the black and magenta arrows indicate the direction of the magnetic field and the orientation of the sample. **b, d** P_r extracted from the *P*-*E* hysteresis loops as a function of in-plane magnetic field at **H** // *S*-direction and **H** ⊥ *S*-direction configurations. **e** Schematics of spiral configuration in top views. **f, g, h** Magnetic control behaviors of **e** vs **q**. The black dashed line indicates the direction of the magnetic field.

Supplementary References

1. Blöchl, P. E. et al. Projector augmented-wave method. *Phys. Rev. B* **50**, 17953-17979 (1994).
2. Kresse, G. et al. From ultrasoft pseudopotentials to the projector augmented-wave method. *Phys. Rev. B* **59**, 1758-1775 (1999).
3. Kresse, G. et al. Efficient iterative schemes for ab initio total-energy calculations using a plane-wave basis set. *Phys. Rev. B* **54**, 11169-11186 (1996).
4. Kresse, G. et al. Efficiency of ab-initio total energy calculations for metals and semiconductors using a plane-wave basis set. *Comp. Mater. Sci.* **6**, 15-50 (1996).
5. Solovyev, I. V. et al. Corrected atomic limit in the local-density approximation and the electronic structure of D impurities in Rb. *Phys. Rev. B* **50**, 16861-16871 (1994).
6. Botana, A. S. et al. Electronic structure and magnetism of transition metal dihalides: bulk to monolayer. *Phys. Rev. Mater.* **3**, 044001 (2019).
7. Grimme, S. et al. Semiempirical GGA-type density functional constructed with a long-range dispersion correction. *J. Comput. Chem.* **27**, 1787-1799 (2006).
8. Perdew, J. P. et al. Generalized gradient approximation made simple. *Phys. Rev. Lett.* **77**, 3865-3868 (1996).
9. Liu, N. S. Competing multiferroic phases in monolayer and few-layer NiI_2. *Phys. Rev. B* **109**, 195422 (2024).
10. Katsura H. et al. Spin current and magnetoelectric effect in noncollinear magnets. *Phys. Rev. Lett.* **29**, 057205 (2005).
11. Zhao, D. et al. Switching dynamics in ferroelectric P(VDF-TrFE) thin films. *Phys. Rev. B* **92**, 214115 (2015).
12. Liu, H. et al. Vapor deposition of magnetic van der Waals NiI_2 crystals. *ACS Nano* **14**, 10544-10551 (2020).
13. Golla, D. et al. Optical thickness determination of hexagonal boron nitride flakes. *Appl. Phys. Lett.* **102**, 161906 (2013).
14. Abhilash, J. J. et al. Ferro-pyroelectric response of 0.57BF-0.31PMN-0.12PT ternary ceramic far away from morphotropic phase boundaries. *Ceram. Int.* **43**, 16676-16683 (2017).
15. Hussain, A. et al. Ferroelectric Sb-doped PMN-PT crystal: high electromechanical response with true-remanent polarization and resistive leakage analyses. *J. Mater. Sci-Mater. El.* **29**, 19567-19577 (2018).
16. Knobloch, T. et al. The performance limits of hexagonal boron nitride as an insulator for scaled CMOS devices based on two-dimensional materials. *Nat. Electron.* **4**, 98-108 (2021).
17. Yang, T. H. et al. Ferroelectric transistors based on shear-transformation-mediated rhombohedral-stacked molybdenum disulfide. *Nat. Electron.* **7**, 29–38 (2024).
