# Supplementary material for: Coexistence of ferroelectricity and antiferroelectricity in 2D van der Waals multiferroic
Source: Nat Commun. 2024 Oct 4;15:8616. doi: 10.1038/s41467-024-53019-5 (PMC11452644; doi:10.1038/s41467-024-53019-5)
Supplement: Supplementary file 2 — Reporting Summary [file 41467_2024_53019_MOESM2_ESM.pdf]

Corresponding author(s): Bo Peng

Lasing Reporting Summary

Nature Research wishes to improve the reproducibility of the work that we publish. This form is intended for publication with all accepted papers reporting claims of lasing and provides structure for consistency and transparency in reporting. Some list items might not apply to an individual manuscript, but all fields must be completed for clarity.

For further information on Nature Research policies, including our data availability policy, see Authors & Referees.

• Experimental design

Please check: are the following details reported in the manuscript?

1. Threshold

Item	Yes	No	Details
Plots of device output power versus pump power over a wide range of values indicating a clear threshold	☐	☒	N/A

2. Linewidth narrowing

Item	Yes	No	Details
Plots of spectral power density for the emission at pump powers below, around, and above the lasing threshold, indicating a clear linewidth narrowing at threshold	☐	☒	N/A
Resolution of the spectrometer used to make spectral measurements	☒	☐	This can be found in Methods.

3. Coherent emission

Item	Yes	No	Details
Measurements of the coherence and/or polarization of the emission	☐	☒	N/A

4. Beam spatial profile

Item	Yes	No	Details
Image and/or measurement of the spatial shape and profile of the emission, showing a well-defined beam above threshold	☐	☒	N/A

5. Operating conditions

Item	Yes	No	Details
Description of the laser and pumping conditions *Continuous-wave, pulsed, temperature of operation*	☒	☐	This can be found in Methods.
Threshold values provided as density values (e.g. W cm^{-2} or J cm^{-2}) taking into account the area of the device	☒	☐	This can be found in Methods.

6. Alternative explanations

Item	Yes	No	Details
Reasoning as to why alternative explanations have been ruled out as responsible for the emission characteristics *e.g. amplified spontaneous, directional scattering; modification of fluorescence spectrum by the cavity*	☐	☒	N/A

7. Theoretical analysis

Item	Yes	No	Details
Theoretical analysis that ensures that the experimental values measured are realistic and reasonable *e.g. laser threshold, linewidth, cavity gain-loss, efficiency*	☐	☒	N/A

8. Statistics

Item	Yes	No	Details
Number of devices fabricated and tested	☒	☐	This can be found in Figure 3a and Supplementary Fig. 8 and 9
Statistical analysis of the device performance and lifetime (time to failure)	☒	☐	This can be found in Figure 4 and Supplementary Fig. 12 and 15

March 2018
